# Multiple-Network Alterations in Major Depressive Disorder With Gastrointestinal Symptoms at Rest Revealed by Global Functional Connectivity Analysis

**DOI:** 10.3389/fnins.2022.897707

**Published:** 2022-06-24

**Authors:** Meiqi Yan, Xiaoya Fu, Yangpan Ou, Feng Liu, Huabing Li, Wenbin Guo

**Affiliations:** ^1^Department of Psychiatry and National Clinical Research Center for Mental Disorders, The Second Xiangya Hospital of Central South University, Changsha, China; ^2^Department of Radiology, Tianjin Medical University General Hospital, Tianjin, China; ^3^Department of Radiology, The Second Xiangya Hospital of Central South University, Changsha, China; ^4^Department of Psychiatry, Qiqihar Medical University, Qiqihar, China; ^5^Department of Psychiatry, The Third People’s Hospital of Foshan, Foshan, China

**Keywords:** global functional connectivity, major depressive disorder, gastrointestinal symptoms, resting state, functional magnetic resonance imaging

## Abstract

**Objective:**

Gastrointestinal (GI) symptoms are prominent in major depressive disorder (MDD) and bring patients lots of complaints and troubles. We aimed to explore whether there were some distinctive brain image alterations in MDD with GI symptoms, which could be used to distinguish MDD with GI symptoms from those without GI symptoms and healthy controls (HCs).

**Methods:**

A total of 35 outpatients with GI symptoms, 17 outpatients without GI symptoms, and 28 HCs were recruited. All the participants were scanned by a resting-state functional magnetic resonance imaging. Imaging data were analyzed with the global functional connectivity (GFC) and support vector machine methods.

**Results:**

MDD with GI symptoms showed decreased GFC in the left superior medial prefrontal cortex (MPFC) compared with MDD without GI symptoms. Compared with HCs, MDD with GI symptoms showed decreased GFC in the bilateral middle temporal pole (MTP) and left posterior cingulate cortex/precuneus (PCC/Pcu), and increased GFC in the right insula and bilateral thalamus. SVM analysis showed that an accuracy was 78.85% in differentiating MDD with GI symptoms from MDD without GI symptoms by using the GFC of the left superior MPFC. A combination of GFC of the left PCC/Pcu and bilateral MTP exhibited the highest accuracy (87.30%) in differentiating patients with MDD with GI symptoms from HCs.

**Conclusion:**

MDD with GI symptoms showed abnormal GFC in multiple networks, including the default mode network and cortico-limbic mood-regulating circuit. Using abnormal GFC might work well to discriminate MDD with GI symptoms from MDD without GI symptoms and HCs.

## Introduction

In addition to the main psychological symptoms (such as persistently depressed mood state), major depressive disorder (MDD) often appears with medically unexplained somatic symptoms (Jain, 2009). Among these, gastrointestinal (GI) symptoms are prominent which bring patients with MDD a lot of complaints and troubles. A literature review (Lépine and Briley, 2004) supported that pain led to depression which in turn increased sensitivity to pain, which indicated that there might be bidirectional influences between MDD and GI symptoms (Karling et al., 2007). Besides, patients with somatic symptoms seemed to undergo more difficult and less effective treatment than patients without somatic symptoms. Some previous studies have shown that somatic symptoms were associated with exacerbation of clinical symptoms, low remission rates (Novick et al., 2013), and poor prognoses (Bekhuis et al., 2016) in patients with MDD. Nevertheless, in outpatient services, general physicians experienced hardly to recognize mental disorders such as MDD, and the chief complaint of somatic symptoms added a more difficult identification (Jain, 2009), which was often referred as to “masked depression” (Verster and Gagiano, 1995). Consequently, patients with MDD with GI symptoms underwent chronic lack of proper diagnoses and effective treatments (Kirmayer et al., 1993; García-Campayo et al., 2008). The economic burden increased because of more healthcare resources utilization during episodes of MDD (Painchault et al., 2014). It would result in an increasing medical cost of MDD and additional waste of medical resources (García-Campayo et al., 2008) when they constantly sought medical help. In the context of a global scarcity of psychiatrists and other mental health resources (Smith, 2014) increased consumption of medical resources caused by the failure to get a correct diagnosis undoubtedly undermines the rational allocation of medical resources. Thus, effective and proper identifications of MDD with GI symptoms are of great significance for the prognosis of MDD and the rational medical resource allocation.

However, the mechanism of GI symptoms in MDD remains unclear. Brain imaging techniques were greatly applied to research the brain image abnormalities in many mental disorders, by using different methods such as gray matter volume (GMV), network homogeneity (NH), functional connectivity (FC), and regional homogeneity (ReHo). Brain structural and functional aberrations were found in patients with MDD with GI symptoms based on literature reviews, such as altered GMV and ReHo (Yan et al., 2019; Liu et al., 2020). But, there is a lack of research into connections between spatially distant brain regions. Current evidence indicated that MDD exhibited abnormal resting-state FC in discrete brain networks, especially in the default-mode network (DMN) and cortico-limbic mood-regulating circuit (MRC) (prefrontal-amygdalar-pallidostriatal-mediothalamic MRC) (Wang et al., 2012). The DMN was regarded as a critical role in gastric sensations (Skrobisz et al., 2020) and functional abnormalities were reported in digestive diseases (Fan et al., 2019). Thus, it was curious to see whether FC of these areas was similarly altered in patients with MDD with GI symptoms. To avoid bias of the pre-selection of seeds in the seed-based regions of interest (ROI) methods and face the controversial issue of the number of independent components in the independent component analysis (ICA) approach (Kelly et al., 2012), a model-free voxel-wised global functional connectivity (GFC) method was applied in the present study with a less biased way (Cui et al., 2018; Ding et al., 2019; Pan et al., 2019).

Taken together, we aimed to explore the GFC differences across patients with MDD with GI symptoms, patients with MDD without GI symptoms and healthy controls (HCs) in the present study. We hypothesized that patients with MDD with GI symptoms would show abnormal GFC in multiple networks, especially in the DMN or cortico-limbic MRC. And it was expected that the altered GFC would correlate with clinical characteristics of patients with MDD with GI symptoms. In addition, the support vector machine (SVM) method was used to detect whether GFC values in these abnormal brain regions might be potential image markers to distinguish patients with MDD with GI symptoms from those without GI symptoms and HCs.

## Materials and Methods

### Participants

A total of 35 outpatients with one or more GI symptoms (GI symptoms group) and 17 outpatients without any GI symptoms (non-GI symptoms group) were recruited. GI symptoms mainly included medically unexplained nausea, vomiting, gastralgia (stomachache), gastric distention, constipation, diarrhea, heartburn, acid reflux. All of them were 18–55 years old outpatients from the Second Xiangya Hospital of Central South University, China. They received their final diagnoses independently by two psychiatrists according to the DSM-5 criteria. Inclusion criteria were: (1) first time of major depressive episode; (2) the 17-item Hamilton Rating Scale for Depression (HRSD-17) (Hamilton, 1967) total scores ≥ 17; (3) with no relative history of medication and physical therapy (such as repetitive transcranial magnetic stimulation, electroconvulsive therapy); (4) with no confirmed digestive tract diseases. The severity of MDD was evaluated by using the HRSD-17. The HRSD-17 can be divided into the following five types of factors: (1) anxiety/somatization (six items of psychic anxiety, somatic anxiety, gastrointestinal symptoms, hypochondriasis, insight, and general symptoms); (2) weight loss (one item); (3) cognitive disturbances (three items of self-guilt, suicide, and agitation); (4) retardation symptoms (four items of depression, work and interests, retardation, and sexual symptoms); (5) sleep disturbances (three items of difficulty falling asleep, superficial sleep, and early awakening).

A total of 28 HCs were recruited from the community, demographically matched (on age, gender, and education) with the patients. Anyone with a suspicious or explicit history of mental disorders running in the family would be excluded, same as any history of digestive tract diseases, neurological diseases, substance abuse, or psychotic symptoms (such as hallucination and delusion, etc.).

All the participants were right-handed and Han Chinese. Exclusion criteria for all the participants are as follows: (1) other mental disorders meeting DSM-5 criteria; (2) with any history of neurological diseases, severe physical illnesses, and substance abuse; (3) be pregnant; (4) with structural abnormalities of the brain; (5) and with any contraindications to MRI scanning.

The study was approved by the Medical Research Ethics Committee of the Second Xiangya Hospital of Central South University, China. It was conducted in accordance with the Helsinki Declaration. Each participant has submitted a written informed consent before enrollment.

### Imaging Acquisition and Data Preprocessing

All the participants underwent a 3.0 T Siemens scanner (Germany) resting-state MRI scan in the Second Xiangya Hospital of Central South University. Initial brain image data were obtained using the echo planar imaging (EPI) sequence. The specific parameters were as follows: repetition time/echo time (TR/TE) 2,000/30 ms, 30 slices, 64 × 64 matrix, 90°flip angle, 24 cm field of view, 4 mm slice thickness, 0.4 mm gap, and 250 volumes lasting for 500 s.

Data preprocessing was finished by using DPABI in Matlab (Yan et al., 2016). The first 10 fMRI images data were discarded to avoid the influence of potential unstable factors, such as unstable initial MRI signals and adaptation time of subjects. Then, slice timing and head motion corrections were performed on the images. The maximum displacement on the *x*, *y*, or *z* axis was 2 mm and the maximum angular rotation was 2°. After that, the corrected images were spatially normalized to the MNI space with 3 mm × 3 mm × 3 mm. Next, the fMRI data were filtered by temporal band-pass (0.01–0.08 Hz) and linearly detrended. Some suspicious pseudo covariates were removed, such as signals from the center region of white matter and region of interest (ROI) based on ventricular seeds, the 24-head motion parameters obtained by the rigid body correction. The global signal was not removed in the present study (Hahamy et al., 2014).

### Global Functional Connectivity Analysis

The specific analysis process was consistent with our previous studies (Cui et al., 2018; Ding et al., 2019; Pan et al., 2019). For each participant, voxel-wise GFC (FC between a given voxel and all the other voxels) was calculated within a gray matter mask in Matlab. The gray matter mask was generated in SPM8 at a threshold of probability > 0.2 (Liu et al., 2015). Pearson correlation coefficients (*r*) between time series of all pairs of voxels were calculated and then converted to *z* values using the Fisher *r*-to-z to transformation (Wang et al., 2015). The GFC of a voxel was the mean coefficient of this given voxel with all the other voxels. By composing the GFC of all voxels, the GFC maps were generated.

### Statistical Analyses

Analysis of variance (ANOVA) was used to compare demographic data (age, years of education) and clinical data (HRSD-17 scores, and five factor scores of HRSD-17) across the three groups. A chi-square test was performed to describe gender distribution and differences in illness duration between the two patient groups were compared by using a two-sample *t*-test. *P* < 0.05 was considered statistically significant.

The frame-wise displacement (FD) value was calculated for each participant (Power et al., 2012). The group differences were identified by performing an analysis of covariance (ANCOVA) in individual whole-brain GFC maps across the three groups, followed by *post hoc t*-tests. Age, gender, years of education, and FD were applied as covariates. The results were false discovery rate (FDR) corrected at *p* < 0.05.

### Correlation Analyses

We extracted the mean *z* values from brain regions with abnormal GFC. The correlations between abnormal GFC values and HRSD-17 scores as well as the five factor scores were assessed by the Pearson’s correlation analysis with a threshold of the Benjamini–Hochberg corrected *p* < 0.05.

### Classification Analyses

Using the SVM method, the LIBSVM software package^1^ was conducted in MATLAB to verify the feasibility of using GFC values of abnormal brain regions to distinguish MDD with GI symptoms from those without GI symptoms and HCs. The approach of “leave-one-out” was applied in the study.

## Results

### Demographic and Clinical Characteristics

No group differences were observed in age, years of education, and gender distribution across the three groups. The two patient groups did not differ in illness durations. The HRSD-17 total and factor scores (excepting weight loss) of the two patient groups were all higher than those of HCs. The GI group showed significantly higher weight loss scores than both non-GI group and HCs, whereas these two groups showed no significant difference in weight loss scores. Besides, the HRSD-17 total scores, factor scores of anxiety/somatization, weight loss, and sleep disturbances of the GI group were higher than those of the non-GI group (Table 1).

**TABLE 1:** Demographic and clinical characteristics of the participants.

	S1 (*n* = 35)	S0 (*n* = 17)	HCs (*n* = 28)	*F‵t* or χ*^2^* value	*Post hoc t*-tests or *p*-values
Age (years)	30.86 ± 6.84	30.29 ± 8.05	30.14 ± 5.00	0.102	0.903^a^
Gender(male/female)	13/22	6/11	14/14	1.377	0.502^b^
Handedness (Right/Left)	35/0	17/0	28/0		
Education(years)	14.51 ± 3.28	12.94 ± 3.46	14.61 ± 2.69	1.797	0.173^a^
Illness duration(months)	6.23 ± 4.63	6.94 ± 3.98		0.544	0.589^c^
HRSD-17 scores	22.69 ± 3.41	20.18 ± 2.67	0.89 ± 0.88	585.979	S1 > S0 > HCs
Anxiety/Somatization	7.31 ± 1.92	6.41 ± 1.66	0.39 ± 0.57	174.531	S1 > S0 > HCs
Weight loss	0.80 ± 0.83	0.06 ± 0.24	0	18.741	S1 > S0, HCs
Cognitive disturbances	3.71 ± 1.78	3.41 ± 1.50	0	64.213	S1, S0 > HCs
Retardation symptoms	6.40 ± 1.42	6.76 ± 1.56	0.18 ± 0.39	253.030	S1, S0 > HCs
Sleep disturbances	4.46 ± 1.42	3.53 ± 1.28	0.32 ± 0.55	103.570	S1 > S0 > HCs

*S1, MDD with GI symptoms; S0, MDD without GI symptoms; HCs, healthy controls; HRSD-17, 17-item Hamilton Rating Scale for Depression.*

*^a^The p-value was obtained by analyses of variance.*

*^b^The p-value was obtained by a chi-square test.*

*^c^The p-value was obtained by two-sample t-tests.*

### Global Functional Connectivity Differences Across Groups

**FIGURE 1:**
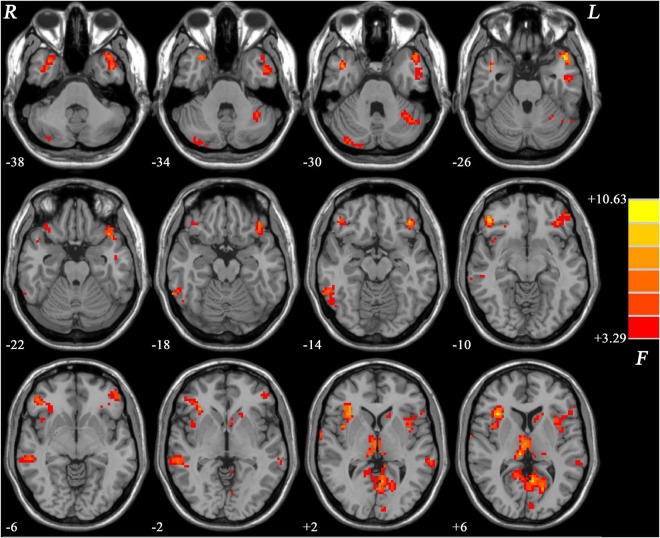
Brain regions showing group differences of GFC values across the three groups. Color bar indicates *F* values from ANCOVA (age, gender, years of education and framewise displacement as covariates). GFC, global functional connectivity; ANCOVA, analysis of covariance.

As shown in Figure 1, the three groups showed significant differences mainly in the frontal, temporal, insular, and limbic areas.

#### Major Depressive Disorder With Gastrointestinal Symptoms Versus Major Depressive Disorder Without Gastrointestinal Symptoms

**FIGURE 2:**
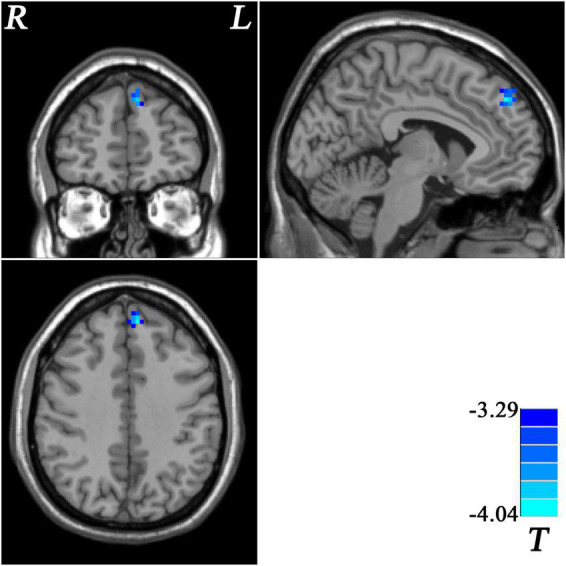
Statistical map depicts lower GFC of MDD patients with GI symptoms compared with MDD patients without GI symptoms. Blue denotes lower GFC. Color bar indicates *T* values from two-sample *t*-test. MDD, major depressive disorder; GFC, global functional connectivity; GI, gastrointestinal symptoms.

Patients with MDD with GI symptoms showed lower GFC in the left superior medial prefrontal cortex (MPFC) than that in patients with MDD without GI symptoms (Table 2 and Figure 2). No higher GFC was observed in the group comparisons.

**TABLE 2:** Significant GFC differences across groups.

Cluster location	Peak (MNI)			Number of voxels	*T*-value
	*x*	*y*	*z*		
*S1* vs. *S0*					
Left Superior MPFC	–6	48	39	41	–4.0430
*S1* vs. *HCs*					
Right Middle Temporal Pole	33	15	–36	27	–4.3420
Left Middle Temporal Pole	–42	18	–24	25	–4.4823
Left Posterior Cingulate Cortex/Precuneus	–6	–60	6	40	–4.1788
Right Insula	33	27	3	23	3.9262
Bilateral Thalamus	9	–27	6	50	4.0570
*S0* vs. *HCs*					
Left Posterior Cingulate Cortex/Precuneus	–3	–63	3	23	–4.0405
Right Inferior Frontal Gyrus	48	39	–9	24	4.6179

*MNI, Montreal Neurological Institute; GFC, global functional connectivity; MPFC, medial prefrontal cortex; GI, gastrointestinal; S1, MDD with GI symptoms; S0, MDD without GI symptoms; HCs, healthy controls.*

#### Major Depressive Disorder With Gastrointestinal Symptoms Versus Healthy Controls

**FIGURE 3:**
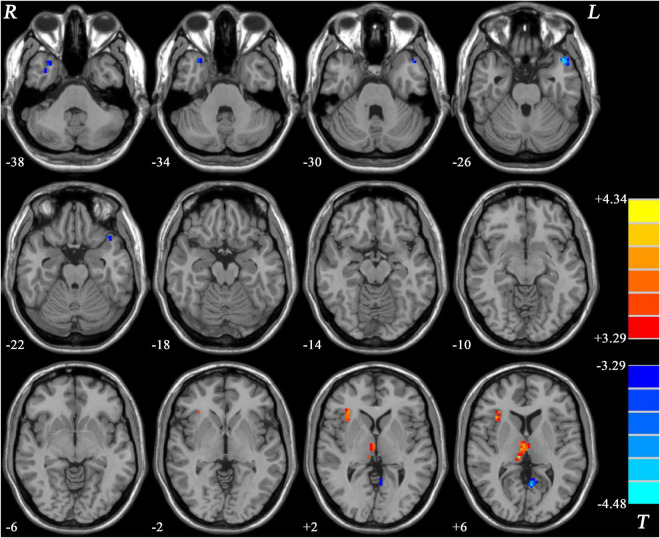
Statistical map depicts higher and lower GFC of MDD patients with GI symptoms compared with healthy controls. Blue denotes lower GFC and red denotes higher GFC. Color bar indicates *T* values from two-sample *t*-test. MDD, major depressive disorder; GFC, global functional connectivity; GI, gastrointestinal symptoms.

Patients with MDD with GI symptoms showed decreased GFC in the bilateral middle temporal pole (MTP) and left posterior cingulate cortex/precuneus (PCC/Pcu), and increased GFC in the right insula and bilateral thalamus, compared with HCs (Table 2 and Figure 3).

#### Major Depressive Disorder Without Gastrointestinal Symptoms Versus Healthy Controls

**FIGURE 4:**
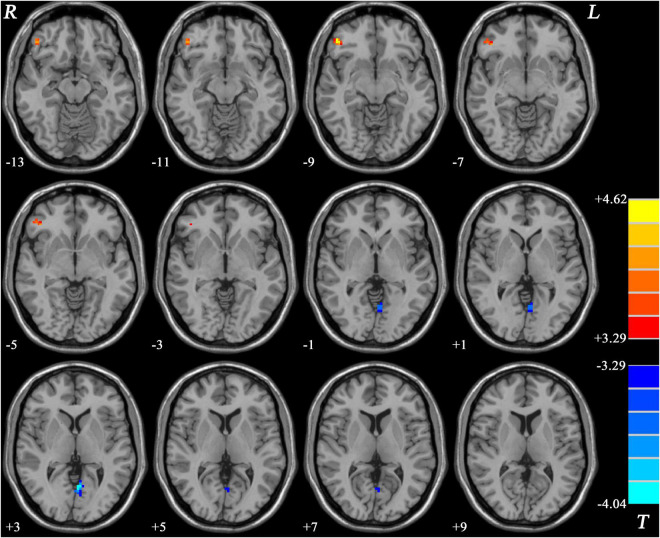
Statistical map depicts higher and lower GFC of MDD patients without GI symptoms compared with healthy controls. Blue denotes lower GFC and red denotes higher GFC. Color bar indicates *T* values from two-sample *t*-test. MDD, major depressive disorder; GFC, global functional connectivity; GI, gastrointestinal symptoms.

Compared with HCs, patients with MDD without GI symptoms showed decreased GFC in the left PCC/Pcu and increased GFC in the right inferior frontal gyrus (IFG) (Table 2 and Figure 4).

### Classification Analyses

#### Major Depressive Disorder With and Without Gastrointestinal Symptoms

**FIGURE 5:**
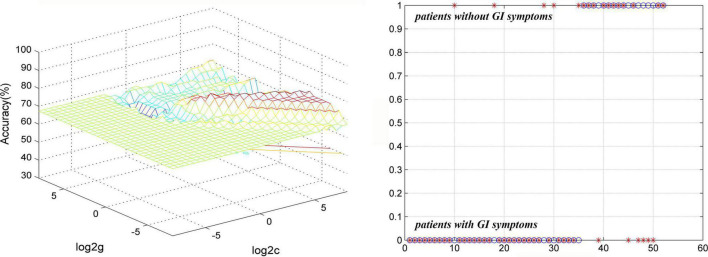
Visualization of classifications through SVM using abnormal GFC values of the left superior MPFC to discriminate MDD with and without GI symptoms. **(Left)** SVM parameters result of 3D view. **(Right)** Classified map of GFC of the left superior MPFC. SVM, support vector machine; GFC, global functional connectivity; MPFC, medial prefrontal gyrus; MDD, major depressive disorder; GI, gastrointestinal symptoms.

The results of SVM analysis showed that the accuracy, sensitivity, and specificity were 78.85% (41/52), 85.71% (30/35), and 64.71% (11/17) respectively, in differentiating patients with MDD with GI symptoms from patients with MDD without GI symptoms by using abnormal GFC values of the left superior MPFC (Figure 5).

#### Major Depressive Disorder With Gastrointestinal Symptoms and Healthy Controls

**FIGURE 6:**
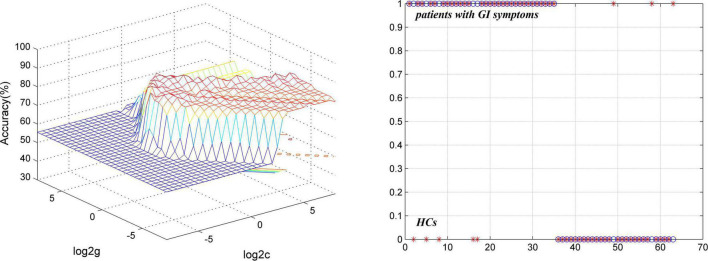
Visualization of classifications through SVM using a combination of GFC values of the left PCC/Pcu and bilateral MTP to discriminate MDD with GI symptoms and HCs. **(Left)** SVM parameters result of 3D view. **(Right)** Classified map of a combination of GFC of the left PCC/Pcu and bilateral MTP. SVM, support vector machine; GFC, global functional connectivity; HCs, healthy controls; PCC/PCu, posterior cingulate cortex/precuneus; MTP, middle temporal pole; MDD, major depressive disorder; GI, gastrointestinal symptoms.

The results of SVM analysis showed that a combination of GFC of the left PCC/Pcu and bilateral MTP exhibited the highest accuracy (87.30%, 55/63), sensitivity (85.71%, 30/35), and specificity (89.29%, 25/28) in differentiating patients with MDD with GI symptoms from HCs (Figure 6).

### Correlations Between Global Functional Connectivity and Clinical Characteristics

For all the patients, GFC of the left superior MPFC was negatively correlated with weight loss scores (*r* = –0.460, *p* = 0.001, the Benjamini–Hochberg correction *p* = 0.006).

For MDD with GI symptoms, GFC of the left superior MPFC was negatively correlated with weight loss scores (*r* = –0.450, *p* = 0.007, the Benjamini–Hochberg correction *p* = 0.021) and positively correlated with retardation symptoms scores (*r* = 0.464, *p* = 0.005, the Benjamini–Hochberg correction *p* = 0.03). GFC in the left MTP was negatively correlated with total HRSD-17 scores (*r* = –0.412, *p* = 0.014, the Benjamini–Hochberg correction *p* = 0.042) and increased GFC of MDD with GI symptoms in the bilateral thalamus was positively correlated with retardation symptoms (*r* = 0.498, *p* = 0.002, the Benjamini–Hochberg correction *p* = 0.012). There were no significant correlations between GFC of the right MTP, right insula, and left PCC/Pcu, and clinical characteristics after the Benjamini–Hochberg correction (see more details in Supplementary Table).

For MDD without GI symptoms, GFC of the left superior MPFC was positively correlated with weight loss scores (*r* = 0.687, *p* = 0.002, the Benjamini–Hochberg correction *p* = 0.012). There were no significant correlations between GFC of both the left PCC/Pcu and right IFG and clinical characteristics after the Benjamini–Hochberg correction (see more details in Supplementary Table).

## Discussion

In this study, the GI group showed more severe depressive symptoms than non-GI group, especially in the terms of anxiety/somatization, weight loss, and sleep disturbances. We observed that GI group showed decreased GFC in the left PCC/Pcu and bilateral MTP and increased GFC in the thalamic and insular areas compared with HCs. And the GI group showed decreased GFC in the left superior MPFC than non-GI group. Good classification results were shown in differentiating GI group from non-GI group and HCs by using SVM analyses.

In addition to the gut microbiota, the brain also regulates food intake through homeostatic control, mood, and cognition (Torres-Fuentes et al., 2017; Milaneschi et al., 2019). Hypothalamus, insula, amygdala, and other limbic and cortical areas participate in the complex processes (Behary and Miras, 2014). During the communication between the central nervous system and peripheral biological signals, the parasympathetic innervation of vagus nerves acts as a vital coordinating role, and vagal nerve blockade may be a surgical therapy in obesity in clinical (Browning et al., 2017). A previous study reported that the cortical neurons that influenced parasympathetic functions were overwhelmingly in the MPFC and insula (Levinthal and Strick, 2020). Thus, dysfunctions in these brain regions may influence body weight by the abovementioned pathways in MDD. In MDD with GI symptoms, function alterations of these brain regions may further affect these processes and lead to weight loss.

Previous studies indicated that somatic symptoms were correlated with more severe clinical symptoms in MDD (Novick et al., 2013) and MDD with GI symptoms showed more severe depression (Liu et al., 2020). Consistent with these studies, the HRSD-17 total scores, factor scores of anxiety/somatization, weight loss and sleep disturbances of GI group were all higher than those of non-GI group which indicated that patients with MDD with GI symptoms showed more severe depressive symptoms than those without GI symptoms. As noted previously, patients with MDD with GI symptoms underwent a prolonged lack of proper diagnoses and experienced lower remission rates and poorer prognoses. Therefore, early detection of patients with MDD with GI symptoms as their chief complaint and aggressive treatments of their depressive symptoms and GI symptoms may help improve the remission rate and positively impact their prognosis.

As one of the core regions of the DMN and part of cortico-limbic MRC, MPFC has attracted a lot of attention in recent years (Raichle, 2015). Involved in a variety of self-referencing, affective, and cognitive functions (such as perceptual memory and executive control) (Lemogne et al., 2012; Schwiedrzik et al., 2018; Lieberman et al., 2019), MPFC performs as a part of the central stress circuit, participating in the process of stress response (Bhatia and Tandon, 2005). In the neuroscience researches, MPFC showed neuroplasticity impairments under chronic stress and depressive-like behaviors (Duman and Aghajanian, 2012; Abdallah et al., 2015; Duman et al., 2016) and similar phenomena existed in researches of cognitive science and clinical psychology level (Price and Duman, 2020). Stress could have both short-term and long-term influences on gastrointestinal tract function and GI symptoms are physical reactions to stressful and emotional conditions (Bhatia and Tandon, 2005). Thus, the function of the MPFC might be associated with depressive symptoms, as well as GI symptoms. At the same time, structural changes in the MPFC may occur with the onset of somatic symptoms, such as gray matter loss in the MPFC, which has been consistently reported to correlate with chronic pain stress (Kang et al., 2019). Previous studies indicated that stress (whatever acute or chronic stress) may induce structural and functional impairments in the MPFC (Bhatia and Tandon, 2005; Wellman and Moench, 2019). These findings suggest that the decrease of GFC in the MPFC in the GI group may be related to the structural changes such as gray matter loss. In addition, portions of the MPFC influenced parasympathetic output to the stomach and related prefrontal neurons were in the left hemisphere more than three times as in the right hemisphere (Levinthal and Strick, 2020), which was consistent with the finding that microstimulation in this region could induce alterations in the gastric function (Hurley-Gius and Neafsey, 1986). Therefore, we speculate that the structure and function of the MPFC may be altered in the vicious cycle of recurrent bad mood and GI symptoms. In the present study, patients with MDD with GI symptoms showed lower GFC in the left superior MPFC than that in patients with MDD without GI symptoms, which may show that decreased GFC in the MPFC might be correlated with the onset of GI symptoms in MDD. Besides, we observed that decreased GFC of MDD with GI symptoms in the left superior MPFC was negatively correlated with weight loss scores and positively correlated with retardation symptoms scores. These further confirmed the important role of the MPFC in MDD with GI symptoms.

A previous study has reported that the volume of the middle temporal gyrus (MTG) was strongly associated with deficits in language and reasoning ability, and both were the state- and trait-related markers of depression (Ang et al., 2020). In our previous studies, we found that melancholic MDD showed lower NH in the bilateral MTG than HCs, especially in the right MTG/MTP (Cui et al., 2017; Yan et al., 2021). Decreased ReHo in the right MTG was also reported in remitted geriatric depression (Yuan et al., 2008). All these findings might reflect the MTG changes as one state characteristic of depression. Consistent with these studies, we observed that patients with MDD with GI symptoms showed decreased GFC in the bilateral MTP compared with HCs in the present study, and decreased GFC in the left MTP was negatively correlated with the severity of depression. Apart from language and memory functions (Squire and Zola-Morgan, 1991; Squire et al., 2004; Baxter, 2009; Friederici and Gierhan, 2013), the temporal lobe has been proposed to be involved in endogenous attention control by one special node and this node was directly connected to the frontal and parietal attention control regions (Sani et al., 2021). PCC has been proposed to be involved in controlling attention by connecting to the frontal lobe (Leech and Sharp, 2014). But whether there is any link between these two regions is unknown. As one of the components of the DMN, PCC/Pcu has attracted much attention in many studies of MDD (Raichle, 2015). Many previous studies have reported decreased activity in the PCC/Pcu in MDD, such as decreased FC, voxel-mirrored homotopic connectivity (VMHC) and ReHo (Bluhm et al., 2009; Chen et al., 2012; Zhu et al., 2012; Guo et al., 2013). Consistently, we observed that both two patient with MDD groups showed decreased GFC in the left PCC/Pcu compared with HCs in the present study. Although it is unclear whether there is a link between the temporal lobe and PCC in attentional control function, we concurrently observed GFC changes in both two brain regions in MDD. Therefore, we speculate that abnormalities in both two brain regions are associated with attention-deficit-induced cognition impairments in MDD.

By direct electrical stimulation of the insula cortex, a variety of visceral responses (containing gastric sensory or motor phenomena, such as nausea, vomiting, belching) and somatic sensation responses were elicited (Penfield and Faulk, 1955; Afif et al., 2010), which promoted the insula to be seen as a primarily visceral–somatic region (Uddin et al., 2017). Besides, cortical neurons that participated in the parasympathetic control of the stomach were overwhelmingly located in the insula (Levinthal and Strick, 2020). Therefore, GI symptoms may occur when the structure or function of the insula were aberrant. For instance, the insula has been commonly found to be involved in the pathophysiologic mechanism of functional dyspepsia (FD) (Van Oudenhove et al., 2010; Zeng et al., 2011; Liu et al., 2018). In addition, the insula has been reported to be associated with abnormal visceral sensitivity in irritable bowel syndrome (IBS) (Icenhour et al., 2017). Thalamus, as the “relay station” of the brain, is recognized as one of the key roles in gastrointestinal sensory processing, because primary interoceptive information is usually afferent to the thalamus, and then it will act as a “gateway” to relay signals to the insula (Zhou et al., 2013; Iannilli et al., 2014; Liu et al., 2018). A previous study observed that patients with FD showed lower connection probability in the right anterior insula with the right thalamus and decreased functional connectivity of them (Liu et al., 2018). Thus, the structure and function of the insula and thalamus may work in the process of the onset and sensory transmission of GI symptoms in MDD. In addition to the “visceral–somatic” functions mentioned earlier, the insula is involved in cognitive control and emotional processes by connection with higher-level cortices and limbic areas (Deen et al., 2011; Chang et al., 2013). Besides, the thalamus, one part of the cortico-limbic MRC, is widely recognized to be involved in the process of attention control, and thus affects cognition (Saalmann and Kastner, 2011; Halassa and Kastner, 2017). And it has been reported that attention deficit was associated with the clinical psychomotor retardation in MDD (Lemelin and Baruch, 1998). To some extent, this is consistent with the findings of the present study that increased GFC in the bilateral thalamus in the GI group was positively correlated with retardation symptoms. Structural and functional changes in the insula and thalamus were reported in many previous studies of MDD (Wise et al., 2017; Peng et al., 2018; Wang et al., 2018; Zhang et al., 2018; Hu et al., 2019). In the present study, we observed that MDD with GI symptoms showed increased GFC in the right insula and bilateral thalamus compared with HCs. And this may account for many of the differences in the clinical manifestations between the GI group and HCs. Reviewing all the aforementioned previous studies, the complexity of the neuropsychiatric system function is well demonstrated.

No less than 60% of the sensitivity or specificity is required for an eligible diagnostic indicator and more than 70% is conducive to the establishment of diagnostic indicators (Swets, 1988; Gong et al., 2011). Thus, results of SVM analysis in differentiating patients with MDD with GI symptoms from those without GI symptoms by using GFC of the left superior MPFC were moderate. And results of SVM analysis in distinguishing patients with MDD with GI symptoms and HCs by using a combination of GFC of the left PCC/Pcu and bilateral MTP were relatively effective. Therefore, we could try to use this method to discriminate MDD with GI symptoms from MDD without GI symptoms and HCs in our clinical work under the dilemma that we can only diagnose through subjective experience and lack objective indicators. However, the clinical symptoms of many patients are complex and there are many confounding factors, so the application scope of this method may be limited.

A previous study indicated that MDD was sensitive to the grading of spectra across resting-state networks (Ries et al., 2018). And Yang et al. (2021) found abnormal FC density in some brain networks in both lower and higher frequency bands in the bipolar disorder during depressive episodes. Thus, whether there are frequency-specific FC changes in MDD with GI symptoms needs further exploration. There are also some limitations. The sample size is small, so the generalization of the research results is limited to some extent. Furthermore, we did not subdivide each of the different GI symptoms because we assumed that they had similar mechanisms. Therefore, we did not know whether different symptoms made different outcomes.

## Conclusion

The present study found that patients with MDD with GI symptoms showed abnormal GFC in multiple networks, including the DMN and cortico-limbic MRC, which suggested that these brain regions may play an important role in the biological mechanisms of GI symptoms in MDD. Abnormal GFC values were correlated with some clinical characteristics. Besides, we observed that MDD with GI symptoms demonstrated more severe depressive symptoms than MDD without GI symptoms. Early identification of patients with MDD with GI symptoms as their chief complaint and aggressive treatments of their depressive symptoms and GI symptoms may help improve the remission rate and positively impact their prognosis. In our clinical practice, it might work well to discriminate MDD with GI symptoms from HCs by using a combination of GFC of the left PCC/Pcu and bilateral MTP.

## Data Availability Statement

The raw data supporting the conclusions of this article will be made available by the authors, without undue reservation.

## Ethics Statement

The studies involving human participants were reviewed and approved by the Second Xiangya Hospital of Central South University, China. The patients/participants provided their written informed consent to participate in this study.

## Author Contributions

MY, XF, and WG contributed to the conception and design of the study. YO, XF, and HL contributed to the data collections. MY, FL, and WG performed the data analysis. MY wrote the manuscript. All authors contributed to manuscript revision, read, and approved the submitted version.

## Conflict of Interest

The authors declare that the research was conducted in the absence of any commercial or financial relationships that could be construed as a potential conflict of interest.

## Publisher’s Note

All claims expressed in this article are solely those of the authors and do not necessarily represent those of their affiliated organizations, or those of the publisher, the editors and the reviewers. Any product that may be evaluated in this article, or claim that may be made by its manufacturer, is not guaranteed or endorsed by the publisher.

## Footnotes

1
http://www.csie.ntu.edu.tw/∼cjlin/libsvm/
