# Supplementary material for: Multiple-Network Alterations in Major Depressive Disorder With Gastrointestinal Symptoms at Rest Revealed by Global Functional Connectivity Analysis
Source: Front Neurosci. 2022 Jun 24;16:897707. doi: 10.3389/fnins.2022.897707 (PMC9263397; doi:10.3389/fnins.2022.897707)
Supplement: Supplementary file 1 [file Table_1.docx]

sTable. Results of r and ﻿ p (two-tailed) values of Pearson correlation analysis between abnormal GFC and clinical characteristics.

| Brain regions |  | Total HRSD-17 | ﻿anxiety/somatization | ﻿weight loss | ﻿cognitive disturbances | ﻿retardation symptoms | ﻿sleep disturbances |
| --- | --- | --- | --- | --- | --- | --- | --- |
| *S0*  Left Superior MPFC  Left PCC/Pcu  Right IFG | r  p  r  p  r  p | 0.174  0.503  -0.070  0.789  -0.131  0.617 | 0.252  0.329  -0.068  0.795  0.088  0.738 | 0.687  0.002**  -0.282  0.272  0.256  0.320 | -0.275  0.286  0.224  0.386  -0.006  0.981 | -0.153  0.558  -0.035  0.893  -0.393  0.119 | 0.416  0.097  -0.225  0.385  0.052  0.843 |
| *S1*  Left Superior MPFC  Right MTP  Left MTP  Left PCC/Pu  Right insula  Bilateral thalamus | r  p  r  p  r  p  r  p  r  p  r  p | -0.067  0.702  -0.274  0.111  -0.412  0.014*  -0.243  0.160  0.335  0.036*  0.367  0.030* | -0,051  0.771  -0.178  0.307  -0.064  0.716  -0.108  0.536  0.184  0.291  0.201  0.247 | -0.450  0.007**  0.089  0.609  0.163  0.348  0.092  0.599  0.033  0.849  -0.318  0.062 | 0.044  0.803  -0.390  0.021*  -0.424  0.011*  -0.180  0.302  0.213  0.220  0.358  0.035* | 0.464  0.005**  -0.173  0.321  -0.371  0.028*  -0,281  0.102  0.246  0.154  0.498  0.002** | -0.346  0.042*  0.189  0.278  -0.099  0.572  0.015  0.931  0.074  0.674  -0.150  0.391 |

MPFC = medial prefrontal cortex, MTP = middle temporal pole, ﻿ PCC/Pcu = posterior cingulate cortex/precuneus, S1=MDD with GI symptoms, S0=MDD without GI symptoms, GI= gastrointestinal.

**correlation is significant at the 0.01level (two-tailed)

*correlation is significant at the 0.05level (two-tailed)
